# Seed-Specific Expression of *Arabidopsis AtCYP85A2* Produces Biologically Active Brassinosteroids Such as Castasterone and Brassinolide to Improve Grain Yield and Quality in Seeds of *Brachypodium Distachyon*

**DOI:** 10.3389/fpls.2021.639508

**Published:** 2021-04-01

**Authors:** Jeehee Roh, Jinyoung Moon, Ye Eun Lee, Chan Ho Park, Seong-Ki Kim

**Affiliations:** ^1^Department of Life Science, Chung-Ang University, Seoul, South Korea; ^2^Department of Plant Biology, Carnegie Institution for Science, Stanford, CA, United States

**Keywords:** seed yield and quality, seed-specific expression, BR 6-oxidase/BL synthase, *Brachypodium distachyon*, *Arabidopsis CYP85A2*

## Abstract

*Brachypodium distachyon* is a monocotyledonous model plant that contains castasterone (CS) but no brassinolide (BL) as the end product of brassinosteroids (BR) biosynthesis, indicating dysfunction of BL synthase, which catalyzes the conversion of CS to BL. To increase BR activity, *Arabidopsis cytochrome P450 85A2* (*AtCYP85A2*) encoding BR 6-oxidase/BL synthase was introduced into *B. distachyon* with the seed-specific promoters *pBSU1, pAt5g10120*, and *pAt5g54000*. RT-PCR analysis and GUS activity revealed that *AtCYP85A2* was only expressed in the seeds of the transgenic plants *pBSU1-AtCYP85A2::Bd21-3, pAt5g10120-AtCYP85A2::Bd21-3*, and *pAt5g54000-AtCYP85A2::Bd21-3*. The crude enzyme prepared from the seeds of these three transgenic plants catalyzed the conversion of 6-deoxoCS to CS. The transgenic plants showed greater specific enzyme activity than the wild-type plant for the conversion of 6-deoxoCS to CS, indicating enhanced BR 6-oxidase activity in the transgenic plants. The enzyme solution also catalyzed the conversion of CS into BL. Additionally, BL was identified from the seeds of transgenic plants, verifying that seed-specific *AtCYP85A2* encodes a functional BL synthase to increase BR activity in the seeds of transgenic Brachypodium. In comparison with wild-type Brachypodium, the transgenic plants showed better growth and development during the vegetative growing stage. The flowers of the transgenic plants were remarkably larger, resulting in increments in the number, size, and height of seeds. The total starch, protein, and lipid contents in transgenic plants were higher than those in wild-type plants, indicating that seed-specific expression of *AtCYP85A2* improves both grain yield and quality in *B. distachyon*.

## Introduction

**Figure 1:**
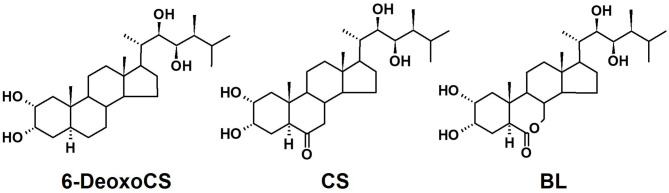
Chemical structure for brassinosteroids mentioned in this study. 6-DeoxoCS, 6-deoxocastasterone; CS, castasterone; BL, brassinolide.

Brassinosteroids (BRs) are steroidal plant hormones that regulate diverse processes in the growth, development, and differentiation of plants (Clouse and Sasse, 1998; Clouse, 2002; Fujioka and Yokota, 2003; Peres et al., 2019). Since brassinolide (BL, Figure 1) was identified from rape pollen, over 50 BRs have been characterized from across the entire plant kingdom (Grove et al., 1979). In bioassays to evaluate BR activity, BL and its direct biosynthetic precursor castasterone (CS) showed stronger activity than other natural BRs, suggesting that BL and/or CS are/is the end products of BR biosynthesis and that other natural BRs may be biosynthetic precursors or catabolites of BL and/or CS in plants (Brosa, 1999; Kim et al., 2004; Joo et al., 2015).

Metabolic studies using biosynthetic intermediates of BR biosynthesis in dicotyledonous plants such as *Catharanthus roseus, Phaseolus vulgaris*, and *Arabidopsis thaliana* have revealed that BL is biosynthesized from campesterol (CR, 24-methylcholesterol), which has the same carbon skeleton as BL, *via* campestanol (CN)-dependent and CN-independent pathways by successive oxidations in the A ring, B ring, and side chain (Bajguz and Tretyn, 2003). Biochemical and molecular genetic analyses of BR-deficient mutants in Arabidopsis, pea, and tomato have characterized the biosynthetic enzymes involved in BR biosynthesis: DET2 as BR 5α-reductase, cytochrome P450 (CYP) 90B1 (DWARF4) as BR C-22 hydroxylase, CYP90A1 as BR C-3 oxidase, CYP90D2 as BR C-23 hydroxylase, CYP85A1 as BR C-6 oxidase, and CYP85A2 as a bifunctional enzyme for BR C-6 oxidase and BL synthase (CS C-7 oxalactonase), thus establishing almost complete pathways for BR biosynthesis in plants. Despite the similarities in BR biosynthesis in dicotyledonous plants, some differences have been noted in monocotyledonous plants (Zhang et al., 2014; Corvalán and Choe, 2017). BL, the most biologically active BR in dicotyledonous plants, has not yet been identified in most monocotyledonous plants. Rice CYP85A1 (OsCYP85A1) and corn CYP85A1 (ZmCYP85A1), homologs of Arabidopsis CYP85A1 (AtCYP85A1) and CYP85A2 (AtCYP85A2), have shown only BR C-6 oxidase activity for the conversion of 6-deoxoCS to CS but not BL synthase activity for the conversion of CS to BL (Mori et al., 2002; Hong et al., 2003; Kim et al., 2005, 2008; Makarevitch et al., 2012). In addition, orthologs of *AtCYP85A2* as BL synthase have not been found in any monocotyledonous plants (Li and Wei, 2020). These findings indicate that in monocotyledonous plants, the most biologically active BR seems to be CS, not BL (Roh et al., 2020).

*Brachypodium distachyon* is a monocotyledonous plant that shows several advantages over monocotyledonous crops. It is a smaller plant, diploid, easily grown, easily crossed, transformable, and has a fully sequenced genome (Draper et al., 2001; Initiative, 2010; Mur et al., 2011; Scholthof et al., 2018). Therefore, it has been proposed as a model plant for investigating monocotyledonous crops, particularly temperate cereals. Recently, a T-DNA-inserted mutant of *bdbrd1-1* (*bdcyp85a1*) that lacked BR C-6 oxidase activity was isolated from *B. distachyon* (Xu et al., 2015). The *bdbrd1-1* mutant plants showed abnormalities in growth and development, including shortened cell shapes, severe dwarfism, twisted leaves, and sterile spikes. Abnormalities in *bdbrd1-1* were restored to the wild-type phenotype by complementation with full-length *BdBRD1*. In addition, BRs exogenously applied to *bdbrd1-1* resulted in partial rescue of the wild-type phenotype. These results indicate that the proper level of endogenous BRs must be maintained for normal growth and development in Brachypodium.

In a recent study, we identified CS but not BL from *B. distachyon* (Roh et al., 2020). The crude enzyme solution prepared from Brachypodium could not catalyze the conversion of CS to BL. Additionally, Brachypodium CYP85A1(BdCYP85A1), a homolog for AtCYP85A1 and AtCYP85A2, showed BR C-6 oxidase activity but did not exhibit BL synthase activity. This indicates that Brachypodium—like rice and corn—cannot produce BL because of the absence of BL synthase, so CS is the end product of BR biosynthesis in the plant. Generally, BL shows 5–10-fold higher biological activity than CS in BR physiology in plants (Kim et al., 2004, 2005; Joo et al., 2015). Vegetative organs such as root, shoot/stem, and leaf in Arabidopsis and tomato show only trace amounts or no BL, but the endogenous levels of BL are higher in reproductive organs such as flowers, seeds, and fruits (Kim et al., 2005; Nomura et al., 2005; Bishop et al., 2006). In Arabidopsis, compared to wild-type plants, plants with overexpressed *AtCYP85A2* (*35S-AtCYP85A2*) yielded larger siliques that contained an increased number of seeds, suggesting that BL synthesized by AtCYP85A2 plays important roles in the growth and development of plants' reproductive organs (Kim et al., 2005). Therefore, introduction of BL synthase to monocotyledonous plants may promote the growth and development of reproductive organs, resulting in improved seed development. However, the progress in the application of BL synthase activity for the breeding of monocotyledonous crops has been delayed by the relatively long lifecycles of these crops and the difficulties in performing molecular genetics in them. In this study, we attempted to introduce *AtCYP85A2* driven by Arabidopsis seed-specific promoters to *Brachypodium distachyon* as a model for monocotyledonous crops to obtain clues for the use of BL synthase activity in crop breeding. In transgenic Brachypodium, seed-specific *AtCYP85A2* increased BR 6-oxidase activity and successfully encoded a functional BL synthase that is absent in Brachypodium, resulting in increased endogenous levels of CS and BL for improved grain yield and quality in plants. Our attempt to induce the heterologous expression of dicotyledonous *CYP85A2* in the seeds of monocotyledonous Brachypodium may be a promising breeding tool for the development of high-quality crops in agriculture.

## Materials and Methods

### Plant Materials and Growth Conditions

*B. distachyon* Bd21-3 was used in this study. Seeds were planted in soil immediately and plants were grown in an environmental growth chamber under a 22°C, 18-h-light (120 μmol photons m^−2^ s^−1^)/20°C, 6-h-dark cycle. The leaves of 2-week-old plants were harvested for RNA extraction. Five-week-old plants were harvested and stored at −80°C until required for endogenous BR analysis and crude enzyme preparation. To dissect the immature embryos of Brachypodium, plants were grown until pollination.

To clone seed-specific promoters and genes for BL synthase, *AtCYP85A2*, Col-0 (*Arabidopsis thaliana*) were both used in this study. *Arabidopsis* seeds were washed in 70% EtOH (v/v) and rinsed with autoclaved sterile water. After cold treatment for 2 days at 4°C, the seeds were planted in a 0.5x Murashige and Skoog (MS) medium containing 1% sucrose and 0.8% plant agar. Plants were grown in an environmental growth chamber at 22°C, with a 16-h-light (120 μmol photons m^−2^ s^−1^)/20°C, 8-h-dark cycle. Two-week-old Arabidopsis plants were used for genomic DNA and RNA extraction.

### Preparation of Seed-Specific *AtCYP85A2* Construct

To produce transgenic Brachypodium, seed-specific *AtCYP85A2* constructs were produced. Three Arabidopsis seed-specific promoters were cloned into the binary vector pCAMBIA1381, which contained multiple cloning sites between the cauliflower mosaic virus (CaMV) *35S* promoter and *GUS* (β*-glucuronidase*). A promoter of *At5g10120* (*pAt5g10120*) and a promoter of *At5g54000* (*pAt5g54000*) were provided by Jeong Sheop Shin at Korea University, Seoul, Republic of Korea (Jeong et al., 2014). The genomic DNA prepared from Col-0 was used to obtain a promoter of *BSU1* (*pBSU1*) and the gene encoding BL synthase, *AtCYP85A2*, was cloned into each vector with seed-specific promoters. Supplementary Table 1 describes the primers used in cloning. With DNA sequencing, the connections between seed-specific promoters and *AtCYP85A2* were all confirmed. These constructs were inserted into *Agrobacterium tumefaciens* LBA4404 by electroporation (Bio-Rad, Hercules, CA, USA). Positive colonies by colony PCR were cultured and used in Agrobacterium-mediated transformation.

### Generation of Transgenic Brachypodium Plants

The transgenic Brachypodium mutants were produced using a modified version of the protocol described by Vogel and Hill (2008). Supplementary Table 2 describes the compositions of all media used in this study. The immature seeds of Bd21-3 were collected immediately after pollination. The seeds' lemma were removed and soaked in a washing solution that included 5% sodium hypochlorite and 0.01% Triton X-100. After being rinsed with autoclaved sterile water, the immature embryos were dissected and transferred to callus induction media (CIM). The transferred embryos were incubated for 3 weeks at 23°C in dark conditions and only the yellowish-grown compact embryogenic calli (CEC) were sub-cultured into fresh CIM. The transferred CEC were then incubated for 3 weeks in the same conditions.

The LBA4404 Agrobacterium strain containing the seed-specific *AtCYP85A2* construct was cultured in 3 mL of LB media containing 75 mg L^−1^ kanamycin and streptomycin for 2 days at 28°C. After sub-culturing the Agrobacterium in 40 mL of LB at 28°C until O.D. 0.8, the Agrobacterium strain was centrifuged at 3,000 rpm for 20 min. The supernatant was discarded and the cells were resuspended in 20 mL of liquid CIM containing 0.01% poloxamer 188 (Sigma, St. Louis, MO, USA) and 200 μM of acetosyringone (Sigma).

This suspension was added into the CEC-collected plates. After gentle shaking for 90 s, the cell suspension and CEC were incubated for 3 min. The cell suspension was then removed with a pipette, and the transformed CEC was dried on autoclaved filter papers for 7 min. The dried CEC was transferred to a co-cultivation medium and incubated for 3 days at 23°C in dark conditions.

The CEC was transferred to CIM containing 40 mg L^−1^ hygromycin and 150 mg L^−1^ ticarcillin disodium/clavulanate potassium (timentin) and incubated for 6 weeks in dark conditions for selection of the transformed CEC. To proceed with the shooty regeneration, the selected CEC was moved to a regeneration medium (ReM) composed of 40 mg L^−1^ hygromycin and 75 mg L^−1^ timentin and incubated for 6 weeks in an environmental growth chamber under a 22°C, 18-h-light (120 μmol photons m^−2^ s^−1^)/20°C, 6-h-dark cycle. The shooty-regenerated CEC was then transferred to the rooting media (RoM) and incubated for 3 weeks under the same conditions as those for shooty-regeneration to undergo rooty-regeneration. The regenerated intact transgenic Brachypodium was planted in soil.

### Histochemical GUS Staining

Histochemical GUS staining was conducted by the previous method (Glazebrook and Weigel, 2002). After samples were fixed in cold 90% acetone for 20 min, the sample was transferred into the staining solution containing 2 mM 5-bromo-4-chloro-3-indolyl-β-D-glucuronic acid (Duchefa, Haarlem, Netherlands) in 50 mM Na_2_HPO_4_ buffer, pH 7.2, 2 mM potassium ferrocyanide, 2 mM potassium ferricyanide, 0.2% Triton X-100 with infiltration under vacuum on ice for 20 min. Then, samples were incubated for 37°C overnight. The samples were transferred to 20, 35, and 50% ethanol and fixed with fixative (50% ethanol, 10% acetic acid, and 5% formaldehyde) in sequence, for 20 min. The samples were washed with 70% ethanol and observed with dissecting (Olympus SZ-PT) microscopes.

### Semi-qPCR and Real-Time Quantitative Reverse Transcription PCR (qRT-PCR) Analysis in Transgenic Brachypodium

To confirm the seed-expression level of *AtCYP85A2* in transgenic plants, the roots, shoots (leaves), and seeds of transgenic plants were harvested, and total RNA extraction was performed with TRI Reagent (Invitrogen, Carlsbad, CA, USA). cDNAs were synthesized from 1 μg of the total RNAs by means of an MMLV-reverse transcription system (Promega, Madison, WI, USA) according to the manufacturer's instructions. PCR of *BdGAPDH* and *AtCYP85A2* was performed using cDNA extracted from the same amount of each tissue as the template. *BdGAPDH* was used as the reference gene in Brachypodium. *BdGAPDH* and *AtCYP85A2* were amplified using 20 thermal cycles (95°C for 10 s, 58°C for 10 s, and 72°C for 10 s).

Total RNA extracted from transgenic Brachypodium was analyzed by qRT-PCR for BR metabolic, signaling and seed size determining genes. Quantitative RT-PCR was performed with the CFX96^TM^ Real-Time PCR Detection system (Bio-Rad) using iQ SYBR Green Supermix (Bio-Rad). The thermal and cycling condition was 95°C for 3 min, followed by 45 cycles of 95°C for 10 s, 50°C for 15 s, and 75°C 15 s. Gene expression levels were normalized to *BdGAPDH*. The primers used for the semi-qPCR and qRT-PCR are described in Supplementary Table 1.

### Crude Enzyme Preparation and Enzyme Assay

The crude enzyme assay was performed using the method described in a previous study (Roh et al., 2020). Grown transgenic Brachypodium seeds were harvested, and the remaining portions (shoot and root) from Brachypodium plants were harvested for use as a control. Ten grams of each plant part were collected and used to prepare the cell-free crude enzyme solution. The crude enzyme solution was quantified by means of a Bradford solution (Bio-Rad) using bovine serum albumin (BSA) as a standard. As the substrate, 5 μg of CS was added to 1 mg of cell-free enzyme solution in the presence of a co-factor (NADPH). Enzyme mixtures were incubated at 37°C for 6 h for the enzyme reaction. Ethyl acetate was used to terminate the enzyme reaction and to extract the BR product (1 mL, three times). The ethyl acetate-soluble fraction was concentrated *in vacuo*, loaded into a Sep-Pak C_18_ cartridge column (1 g, Waters Co., Milford, MA, USA), and eluted in 5 mL of 50, 70, 90, and 100% MeOH. The 90% MeOH fractions were dried *in vacuo* and dissolved in a small amount of MeOH to subject them to reverse-phase HPLC (SenshuPak C_18_, 10 × 150 mm). The column was eluted at a flow rate of 2.5 mL min^−1^ using MeCN-water gradients: 0–20 min, 45% MeCN; 20–40 min, 45–100% MeCN gradient; 40–70 min, 100% MeCN. Fractions were collected every minute. To gather the bioactive fractions, rice lamina inclination assay was performed as described in a previous study (Roh et al., 2020). Using authentic BRs, HPLC was performed to double-check the bioactive fractions. Under the same HPLC conditions, authentic BL and CS were detected on 13/14, and 22/23, respectively. The fractions for BL were dried, combined, and analyzed by capillary GC-MS analysis with bismethaneboronation.

### Quantification of Endogenous BRs in Brachypodium Seeds

Endogenous BR analysis was performed in the seeds of the transgenic Brachypodium, *pBSU1-AtCYP85A2::Bd21-3, pAt5g10120-AtCYP85A2::Bd21-3*, and *pAt5g54000-AtCYP85A2::Bd21-3*. Well-grown transgenic Brachypodium and Bd21-3 were harvested and divided into seed and non-seed parts. Fifteen grams of each part were homogenized and purified as described in a previous study (Roh et al., 2020). After purification by reverse-phase HPLC (SenshuPak C_18_) as described above, the fractions for CS and BL were combined and analyzed by capillary GC-selected ion monitoring (SIM)/MS analysis with bismethaneboronation.

### GC-MS Analysis

The GC-MS analyses were performed on a Hewlett-Packard 5973 mass spectrometer (electron impact ionization, 70 electron voltage; Agilent, Santa Clara, CA, USA) connected to a 6890 gas–chromatography fitted with a fused silica capillary column (HP-5, 0.25 mm × 15 m, 0.25-μm film thickness; Agilent). Helium was used as the carrier gas at a flow rate of 1 mL min^−1^, and samples were introduced using an on-column injection mode. The initial oven temperature was 175°C and maintained for 2 min, elevated to 280°C at a rate of 40°C min^−1^, and then maintained at 280°C for 15 min. Methaneboronation was performed by heating samples dissolved in pyridine containing methaneboronic acid (2 mg mL^−1^; Sigma) at 80°C for 20 min.

### Quantification of Total Soluble Starch, Protein, and Lipid Contents in Brachypodium Seeds

The measurement of endogenous starch content in transgenic Brachypodium seeds was performed using the Starch Assay Kit (Abcam, Cambridge, United Kingdom) according to the manufacturer's instructions. After color development of each sample, their absorbances were measured at 570 nm. The standard curve was constructed using the starch standard included in the kit.

Quantification of total protein content was performed with a modified version of the method employed by Focks and Benning (1998). Seeds were ground in liquid nitrogen and 250 μL of cold acetone was added to each sample. The samples were incubated at −20°C for 10 min to precipitate proteins. After incubation, the samples were centrifuged at 16,000 × *g* for 20 min at 4°C, and the supernatant was discarded by means of suction. After a brief period of drying, the pellets were resuspended into 250 μL of total protein extraction buffer (50 mM Tris-HCl, 250 mM NaCl, 1 mM EDTA, 1% SDS [w/v]). These samples were incubated at room temperature (RT) for 2 h with gentle shaking and then centrifuged at 16,000 × *g* for 5 min at 4°C. The supernatant was measured by the Bradford solution (Bio-Rad) at 595 nm. Bovine serum albumin (BSA) was used as a standard to generate a standard curve.

For quantifications of total lipid content in seeds, a modified version of the method employed by Mishra was used (Mishra et al., 2014). Seeds were ground in liquid nitrogen and 600 μL of CHCl_3_/MeOH (in a 2:1 ratio) was added to each sample. After mixing by vortex for 2 min, this mixture was centrifuged at 13,000 rpm for 15 min. The supernatants were collected, and the process was repeated three times. The supernatant was dried in a vacuum at 50°C. A vanillin-phosphoric acid reagent (SPV reagent, 0.2 mg mL^−1^ vanillin, 17% phosphoric acid) was prepared and stored in dark conditions. The samples were then transferred to a test tube and resuspended in sterile water. After incubation of the tubes at 100°C for 10 min, the tubes were cooled for 5 min in ice. Five milliliters of SPV reagent was added to each sample and incubated by means of shaking at 200 rpm and 37°C for 15 min. When color development appeared, the absorbance was measured at 530 nm. Cholesterol (CHR) was used as the standard to produce the standard curve.

### Statistical Analysis

For phenotype analysis in this study, Brachypodium seeds were visually examined. For the measurement of the length and width, 200 observations were made for each line. Brachypodium seeds were weighed in five sets of 10 units (50 units per experiment) with 10 replicates (total 500 seeds for each line).

Student's *t*-test was used to analyze the data for each group and the normality of all data was assessed using the Shapiro–Wilk test. For multiple comparisons, the data were analyzed by one-way analysis of variance (ANOVA) with the Holm–Sidak method.

## Results

### Generation of Seed-Specific *AtCYP85A2-*Expressing Transgenic Brachypodium

**Figure 2:**
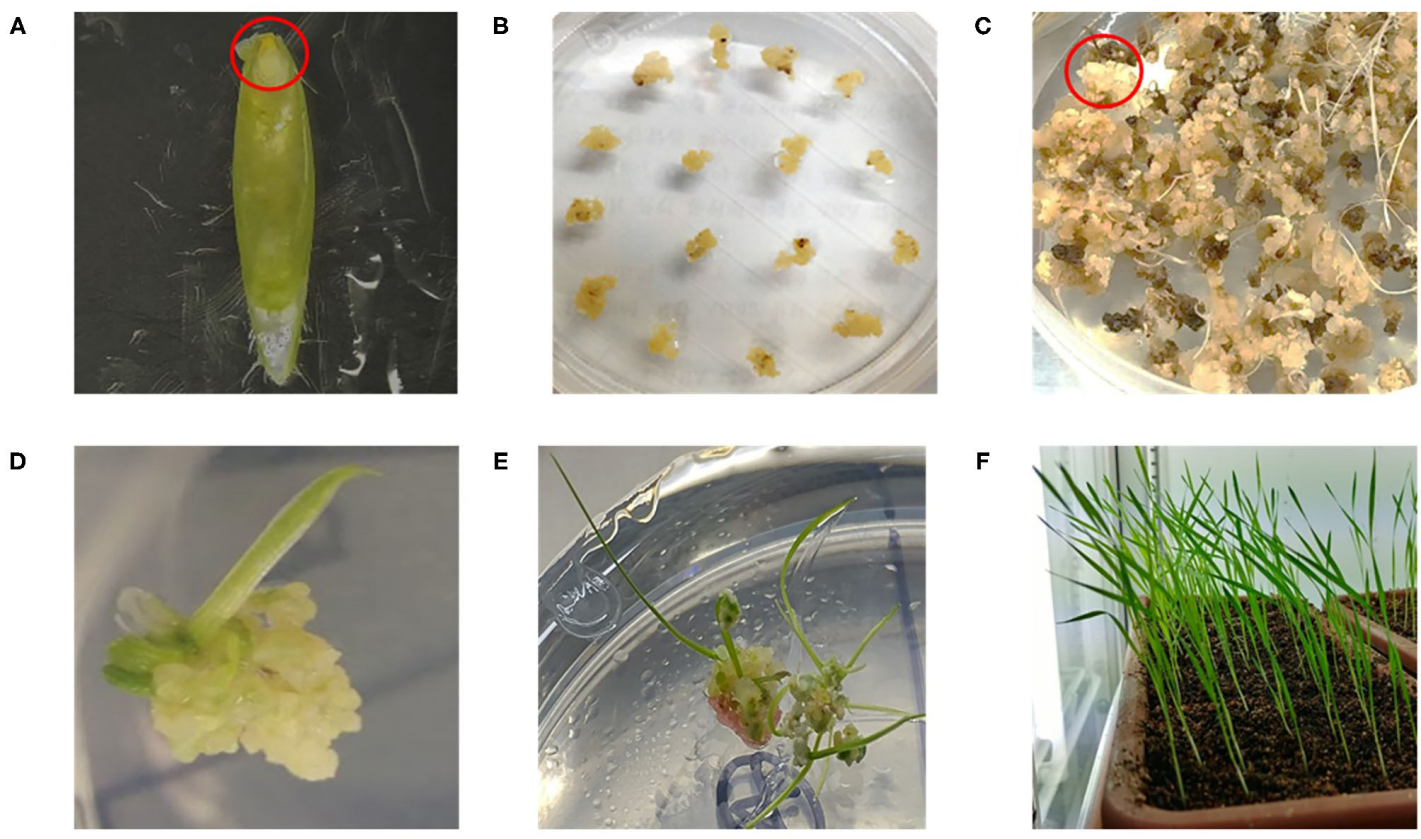
Preparation of seed-specific *AtCYP85A2-*expressing transgenic *Brachypodium***.** **(A)** Region of embryonic cells (red circle) dissecting from immature seed of Bd21-3, the wild type Brachypodium. **(B)** Calli induced from embryonic cells in callus induction media (CIM). **(C)** Hygromycin resistant yellowish-brown calli after agrobacterium-mediated transformation. Red circle indicates the selected calli. **(D)** Regenerated shoot from *AtCYP85A2*-expressing callus in regeneration medium (ReM) for 7 days. **(E)** Regenerated shoot and root from *AtCYP85A2*-expressing callus in ReM for 21 days. **(F)** *AtCYP85A2*-expressing transgenic Brachypodium plants.

**Figure 3:**
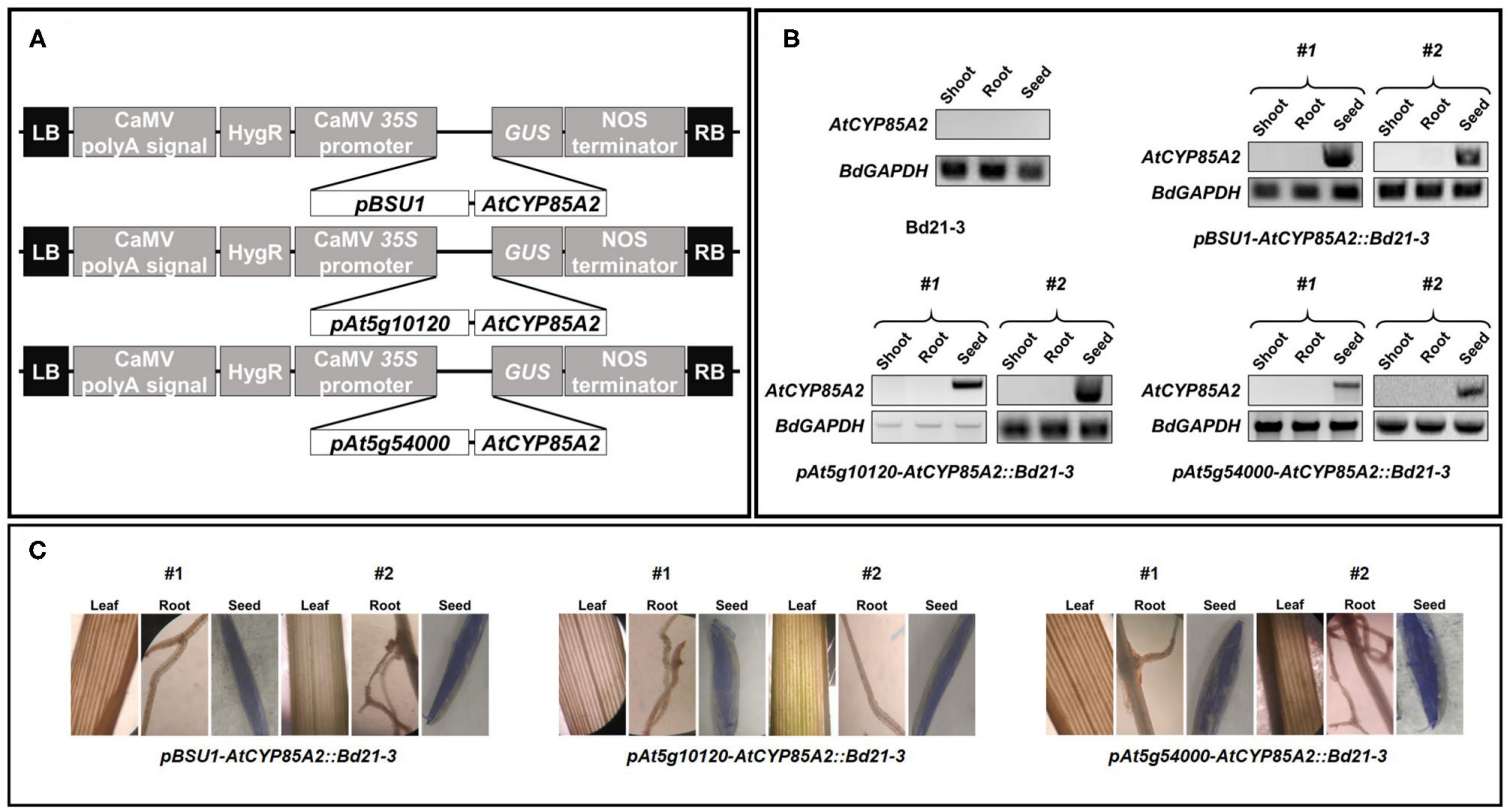
Genetic map of seed-specific *AtCYP85A2-*expressing constructs and expression level of *AtCYP85A2* in transgenic Brachypodium mutants. **(A)** Genetic map of *AtCYP85A2* expressing constructs driven by seed specific promoters, *pBSU1, pAt5g10120*, and *pAt5g54000*. CaMV polyA signal, the cauliflower mosaic virus polyadenylation signal; HygR, hygromycin resistance gene; CaMV *35S* promoter, the cauliflower mosaic virus *35S* promoter; *GUS*, β*-glucuronidase* gene; NOS terminator, nopaline synthase terminator; LB, left border; RB, right border. **(B)** Semi-quantitative RT-PCR analysis for expression of *AtCYP85A2* in shoot, root, and seed of wild type and transgenic plants. #1 and #2 represent two different lines for three transgenic plants. *AtCYP85A2* was only detected in seed of transgenic plants. *BdGAPDH* is a reference gene. **(C)** GUS-staining in leaf, root and seed of transgenic Brachypodium mutants. GUS activity was concentrated in seed of transgenic plants.

Embryonic cells obtained from immature seeds of *Brachypodium distachyon* (Figure 2A) were collected and transferred to agar plates containing callus induction media (CIM), as summarized in Supplementary Table 2 (Figure 2B). To produce transgenic Brachypodium, three Arabidopsis seed-specific promoters, *pBSU1, pAt5g10120*, and *pAt5g54000* were cloned into the binary vector. The gene *AtCYP85A2* was then cloned into the vectors containing *pBSU1, pAt5g10120*, or *pAt5g54000* (Figure 3A). The resulting constructs were transferred into *Agrobacterium tumefaciens*, and antibiotic-resistant colonies were collected and cultured. Yellowish-brown calli with *AtCYP85A2* driven by *pBSU1, pAt5g10120*, and *pAt5g54000* were selected (Figure 2C) and moved to regeneration media for shoot formation. The shoot-regenerated callus was then transferred to the root regeneration media, resulting in both shoot- and root-regenerated calli containing the seed-specific *AtCYP85A2* constructs (Figures 2D,E). The obtained regenerated calli were planted in soil, and the seed-specific *AtCYP85A2*-expressing transgenic Brachypodium plants *pBSU1-AtCYP85A2::Bd21-3* (#1 and #2)*, pAt5g10120-AtCYP85A2::Bd21-3* (#1 and #2), and *pAt5g54000-AtCYP85A2::Bd21-3* (#1 and #2) were prepared (Figure 2F). Semi-qRT-PCR analysis for expression of *AtCYP85A2* in transgenic Brachypodium plants showed that *AtCYP85A2* is strongly expressed in the seed but not in the shoot (the aerial part without seed) or root (Figure 3B). Additionally, GUS activity was concentrated in the seeds of transgenic plants (Figure 3C), verifying that *AtCYP85A2* is seed-specifically introduced in *pBSU1-AtCYP85A2::Bd21-3, pAt5g10120-AtCYP85A2::Bd21-3*, and *pAt5g54000-AtCYP85A2::Bd21-3* transgenic Brachypodium.

### Seed-Specific Expression of *AtCYP85A2* Produces Active BRs in Seeds of Transgenic Brachypodium

Quantitative analyses for 6-deoxoCS, CS, and BL were performed in the seeds of *Bd21-3, pBSU1-AtCYP85A2::Bd21-3, pAt5g10120-AtCYP85A2::Bd21-3*, and *pAt5g54000-AtCYP85A2::Bd21-3*. After adding [26. 28-^2^H_6_- 6-deoxoCS], [26. 28-^2^H_3_-CS], and [26. 28-^2^H_3_-BL] for quantitative analysis, ethyl acetate soluble fractions obtained from the seeds of Brachypodium were purified by silica gel and C_18_ column chromatography followed by reverse-phase HPLC. The active HPLC fractions corresponding to synthetic 6-deoxoCS (fraction 40–42), CS (fraction 22/23), and BL (fraction 13/14) were collected and analyzed by capillary GC-MS/SIM after methaneboronation. Endogenous amounts of 6-deoxoCS, CS, and BL in the seeds of Brachypodium were calculated by determining the ratio of endogenous BRs/the [^2^H_3_] or [^2^H_6_] labeled BRs added as internal standards.

As summarized in Table 1, a bismethaneboronate (BMB) of the active compound in HPLC fraction 40–42 yielded a molecular ion at *m/z* 498 [M^+^] and prominent ions at *m/z* 483, 343, 273, and 155. The MS spectrum and GC retention time were identical to those of authentic 6-deoxoCS BMB, which demonstrates the presence of 6-deoxoCS in seeds of both wild-type and transgenic lines. The endogenous levels of 6-deoxoCS in the seeds of wild-type *Bd21-3, pBSU1-AtCYP85A2::Bd21-3, pAt5g10120-AtCYP85A2::Bd21-3*, and *pAt5g54000-AtCYP85A2::Bd21-3* were 5.29, 2.46, 3.73, and 1.48 ng g^−1^ fresh weight, respectively. In the HPLC fraction 22/23, the BMB of the active compound showed the same MS spectrum at *m/z* 512 [M^+^], 357, 327, 287, and 155 at the same retention time on GC as those of synthetic CS BMB, verifying that CS is present in the seeds of wild-type and transgenic plants. The endogenous levels of CS in the seeds of *Bd21-3, pBSU1-AtCYP85A2::Bd21-3, pAt5g10120-AtCYP85A2::Bd21-3*, and *pAt5g54000-AtCYP85A2::Bd21-3* were 1.55, 1.83, 1.79, and 1.97 ng g^−1^ fresh weight, respectively. While the BMB of the active principle in HPLC fraction 13/14 obtained from the seeds of transgenic Brachypodium exhibited the same MS spectrum (at *m/z* 528[M^+^], 374, 332, 177, and 155) and GC retention time as those of authentic BL BMB, no BL was identified from the wild-type seeds. The endogenous levels of BL in the seeds of *pBSU1-AtCYP85A2::Bd21-3, pAt5g10120-AtCYP85A2::Bd21-3*, and *pAt5g54000-AtCYP85A2::Bd21-3* were calculated as 2.55, 2.24, and 4.19 ng g^−1^ fresh weight. Endogenous levels of both CS and BL were higher than those in the wild type, demonstrating that the seed-specific expression of *AtCYP85A2* can produce more biologically active BRs, CS, and BL in the seeds of transgenic Brachypodium.

**Table 1:** Quantification of endogenous BRs (6-deoxoCS, CS, and BL) in seeds of Brachypodium.

**Seeds of Brachypodium**	**Rt^**b**^ on HPLC**	**RRt^**c**^ on GC**	**Prominent ions (*m/z*, relative intensity %)**	**Endogenous amount^**d**^**
**Compound**				
**Bd21-3**				
6-DeoxoCS^a^	40–42	0.735	498(M^+^, 48), 483(14), 343(7), 273(100), 155(83)	5.29 (0.87)
CS^a^	22/23	1.000	512(M^+^, 12), 357(24), 327(21), 287(42), 155(100)	1.55 (0.26)
BL^a^	N.D.^e^	N.D.^e^	N.D.^e^	N.D.^e^
***pBSU1-AtCYP85A2::Bd21-3***				
6-DeoxoCS^a^	40–42	0.735	498(M^+^, 52), 483(18), 343(11), 273(100), 155(91)	2.46 (0.48)
CS^a^	22/23	1.000	512(M^+^, 8), 357(17), 327(14), 287(35), 155(100)	1.83 (0.33)
BL^a^	13/14	1.300	528(M^+^, 6), 374(33), 332(49), 177(71), 155(100)	2.55 (0.57)
***pAt5g10120-AtCYP85A2::Bd21-3***				
6-DeoxoCS^a^	40–42	0.735	498(M^+^, 54), 483(20), 343(16), 273(100), 155(94)	3.73 (0.73)
CS^a^	22/23	1.000	512(M^+^, 9), 357(21), 327(14), 287(37), 155(100)	1.79 (0.31)
BL^a^	13/14	1.300	528(M^+^, 3), 374(29), 332(42), 177(69), 155(100)	2.24 (0.42)
***pAt5g54000-AtCYP85A2::Bd21-3***				
6-DeoxoCS^a^	40–42	0.735	498(M^+^, 50), 483(17), 343(10), 273(100), 155(89)	1.48 (0.25)
CS^a^	22/23	1.000	512(M^+^, 10), 357(20), 327(16), 287(36), 155(100)	1.97 (0.41)
BL^a^	13/14	1.300	528(M^+^, 5), 374(32), 332(47), 177(72), 155(100)	4.19 (0.98)
Authentic 6-DeoxoCS^a^	40–42	0.735	498(M^+^, 50), 483(17), 343(10), 273(100), 155(89)	-
Authentic CS^a^	22/23	1.000	512(M^+^, 11), 357(21), 327(19), 287(36), 155(100)	-
Authentic BL^a^	13/14	1.300	528(M^+^, 5), 374(31), 332(42), 177(74), 155(100)	-

a *The sample was analyzed as a bismethanboronate in GC-MS/SIM analysis*.

b *Rt : Retention time (min)*.

c *RRt : Relative retention time to CS (15.01 min)*.

d *Amount is denoted as ng g^−1^ fresh weight. Values represent average amount from three replicates. Numbers in parentheses indicate standard error (S.E.)*.

e *Not detected*.

### Seed-Specific Expression of *AtCYP85A2* Enhances BR 6-Oxidase Activity and Newly Encodes BL Synthase in the Seeds of Transgenic Brachypodium

**Figure 4:**
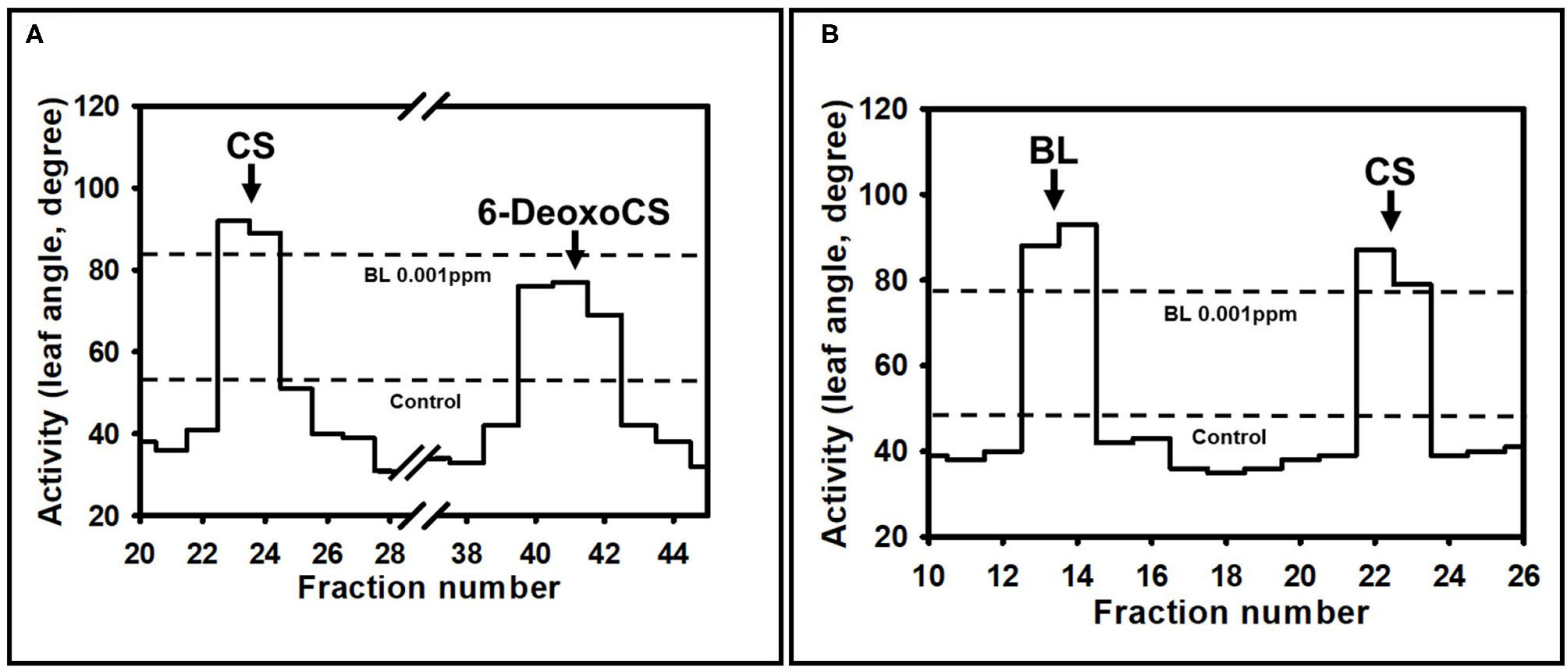
Distribution of BR activity after a reversed phase HPLC. HPLC was carried out using a C_18_ column (SenshuPak, 10 × 150 mm) at flow 2.5 mL min^−1^ using MeCN-water gradients: 0–20 min, 45% MeCN; 20–40 min, 45–100% MeCN gradient; 40–70 min, 100% MeCN. Fractions were collected every minute. The arrow indicates the elution points of authentic BRs. Activities shown were measured by rice lamina inclination assay. The average of 20 rice segments was calculated in every fraction. The dotted line represents the average biological activity after application of no BRs (Control) and BL 0.001 ppm. **(A)** The biological activity after HPLC when 6-deoxoCS was used as a substrate. **(B)** The biological activity after HPLC when CS was used as substrate.

To confirm the RT-PCR findings for *AtCYP85A2* expression and quantitative analysis of endogenous BRs, crude enzyme solutions were prepared from the shoot/root (plants without seeds) and seeds of *Bd21-3, pBSU1-AtCYP85A2::Bd21-3, pAt5g10120-AtCYP85A2::Bd21-3*, and *pAt5g54000-AtCYP85A2::Bd21-3*, and the *in vitro* conversion of 6-deoxoCS to CS and CS to BL in the organs was examined. After termination of enzyme assays, [26. 28-^2^H_3_-CS] and [26. 28-^2^H_3_-BL] were added to enzyme assay mixtures and the product was extracted with ethyl acetate. The ethyl acetate soluble fraction was concentrated and purified by a C_18_ cartridge column. The active fractions in the rice lamina inclination bioassay were combined and further purified by a reverse-phase HPLC. When 6-deoxoCS was used as a substrate, the HPLC fractions 22/23 and 40–42 exhibited BR activity (Figure 4A; Supplementary Figure 1A). Under the same HPLC conditions, authentic 6-deoxoCS was eluted in fraction 40–42, indicating that HPLC fraction 22/23 contained the enzyme product. When CS was added as a substrate, the HPLC fractions 13/14 and 22/23 showed activity for BRs (Figure 4B; Supplementary Figure 1B). Authentic CS was eluted in the HPLC fraction 22/23, indicating that the product was likely contained in fraction 13/14. The active compound in HPLC fraction 22/23 for conversion of 6-deoxoCS to CS and in 13/14 for conversion of CS to BL was derivatized to be a BMB and analyzed by capillary GC-selected ion monitoring (SIM).

Conversion of 6-deoxoCS to CS occurred in all enzyme solutions prepared from shoot/root and seeds of *Bd21-3, pBSU1-AtCYP85A2::Bd21-3, pAt5g10120-AtCYP85A2::Bd21-3*, and *pAt5g54000-AtCYP85A2::Bd21-3*, which suggests that BR 6-oxidase catalyzing the conversion of 6-deoxoCS to CS was functional in all organs of the wild-type and three transgenic Brachypodium (Table 2). The specific enzyme activity of BR 6-oxidase in the shoot/root of the three transgenic Brachypodium was similar to that in the wild type. However, BR 6-oxidase activity in the seeds of transgenic Brachypodium was significantly greater than that in wild-type seeds. Thus, the seed-specific expressed *AtCYP85A2* enhanced BR 6-oxidase activity in the seeds of the three transgenic Brachypodium. Conversion of CS to BL was not demonstrated in the enzyme solutions prepared from shoot/root or seeds of Bd21-3, to which no *AtCYP85A2* had been introduced. Similarly, the enzyme solutions prepared from the shoot/root of *pBSU1-AtCYP85A2::Bd21-3, pAt5g10120-AtCYP85A2::Bd21-3*, and *pAt5g54000-AtCYP85A2::Bd21-3* did not catalyze the conversion of CS to BL either. In contrast, the enzyme preparations from the seeds of the three transgenic Brachypodium successfully catalyzed the conversion of CS to BL. The specific enzyme activities of the BL synthase mediating the conversion of CS to BL in the seeds of *pBSU1-AtCYP85A2::Bd21-3, pAt5g10120-AtCYP85A2::Bd21-3*, and *pAt5g54000-AtCYP85A2::Bd21-3* were 1.14, 0.60, and 1.59 ng mg^−1^ protein min^−1^, respectively. Thus, the heterologous expression of *AtCYP85A2* driven by seed-specific promoters only encodes a functional BL synthase in the seeds of transgenic Brachypodium.

**Table 2:** Conversion of 6-deoxoCS and CS by the crude enzyme solution prepared from Brachypodium.

**Brachypodium**	**Substrate**	**Product^**a**^**	**Specific enzyme activity^**b**^**
**Bd21-3**			
Shoot/Root	6-DeoxoCS	CS	1.70 (0.32)
	CS	N.D.^c^	-
Seed	6-DeoxoCS	CS	1.58 (0.39)
	CS	N.D.^c^	-
***pBSU1-AtCYP85A2::Bd21-3***			
Shoot/Root	6-DeoxoCS	CS	1.80 (0.48)
	CS	N.D.^c^	-
Seed	6-DeoxoCS	CS	1.90 (0.57)
	CS	BL	1.14 (0.26)
***pAt5g10120-AtCYP85A2::Bd21-3***			
Shoot/Root	6-DeoxoCS	CS	1.75 (0.41)
	CS	N.D.^c^	-
Seed	6-DeoxoCS	CS	1.83 (0.42)
	CS	BL	0.60 (0.11)
***pAt5g54000-AtCYP85A2::Bd21-3***			
Shoot/Root	6-DeoxoCS	CS	1.82 (0.43)
	CS	N.D.^c^	-
Seed	6-DeoxoCS	CS	2.03 (0.62)
	CS	BL	1.59 (0.27)

a *The sample was analyzed as a bismethanboronate in GC-MS/SIM analysis*.

b *Activity is denoted as ng mg^−1^ protein min^−1^. Values represent average activity from three replicates. Numbers in parentheses indicate standard error (S.E.)*.

c *Not Detected*.

### Seed-Specific Expression of *AtCYP85A2* Alters Expression of Metabolic Genes in Transgenic Brachypodium

**Figure 5:**
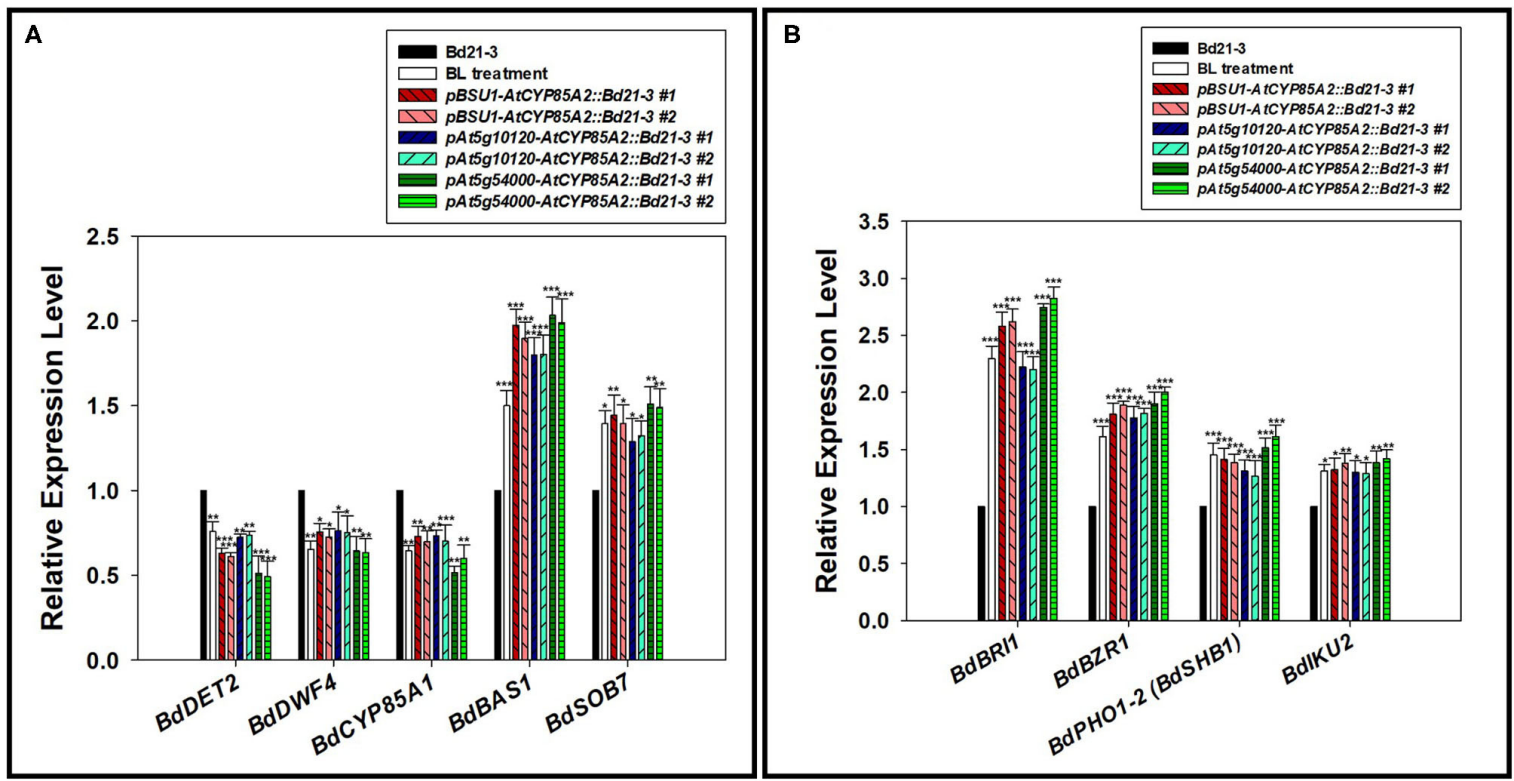
Expression of BR related and seed size determination genes in transgenic Brachypodium mutants. **(A)** Semi-qPCR of BR metabolic genes. *BdDET2(Bradi2g55110), BdDWF4(Bradi1g69040)*, and *BdCYP85A1(Bradi1g15030)* for BR biosynthesis. *BdBAS1(Bradi2g04660)* and *BdSOB7(Bradi2g44140)* for BR catabolism. **(B)** Semi-qPCR of BR signaling and seed size-determining genes. *BdBRI1(Bradi2g48280)* and *BdBZR1(Bradi1g23550)* for BR signaling. *BdPHO1-2 (BdSHB1, Bradi3g54920)* and *BdIKU2(Bradi4g11740)* for seed size determination. #1 and #2 represent two different lines for three transgenic plants. Semi-qRT-PCR was performed with total RNA. *BdGAPDH* was used to normalize the expression level. Two biological repeats along with three technical repeats were performed for quantification of gene expression level. The asterisks indicate the statistical significance of the Student's *t*-test: * (*P* < 0.05), ** (*P* < 0.01), and *** (*P* < 0.001).

Regulation of biosynthetic and catabolic genes by the end product in BR biosynthesis is important to maintain the steady-state level of active BRs in plants (Tanaka et al., 2005; Chung and Choe, 2013; Wei and Li, 2020). We had demonstrated that BdDET2 and BdDWARF4 showed enzyme activity for 5α-reductase and C-22 hydroxylase, respectively, in Brachypodium BR biosynthesis (Roh et al., 2020). As shown in Figure 5A, the expressions of *BdDET2* and *BdDWARF4* were down-regulated in transgenic Brachypodium plants, similar to the findings after exogenous application of BL to Brachypodium. We recently found that both *BdBAS1* (*BdCYP734A6*) and *BdSOB7* (*BdCYP72A15*) show 26-hydroxylase activity in BR catabolism (data will be published elsewhere). In transgenic Brachypodium plants, the expression of both catabolic genes was clearly activated in comparison with that in the wild type, similar to the expression in plants that receive exogenously applied BL. These findings indicate that the feedback regulation of upstream biosynthetic genes and feedforward regulation of downstream catabolic genes will likely be mediated by the newly synthesized BL to maintain a steady-state level of the biologically active BR in transgenic Brachypodium.

### Seed-Specific Expression of *AtCYP85A2* Activates Signaling to Promote Seed Development in Transgenic Brachypodium

*BdBRI1(Bradi2g48280)* and *BdBZR1(Bradi1g23550)* in the Brachypodium genome are homologs for *AtBRI1* and *AtBZR1/AtBES1* in *Arabidopsis*, respectively, implying that they may act as a receptor and transcription factor in BR signaling in *B. distachyon*. In the seeds of three transgenic Brachypodium mutants, the expressions of *BdBRI1* and *BdBZR1* were significantly enhanced in comparison with those in the wild type (Figure 5B). Coupled with the finding that exogenously applied BL enhances the expression of both genes, this result indicates that the BL newly synthesized by the heterologously expressed *AtCYP85A2* triggers the activation of BR signaling in the seeds of transgenic Brachypodium. In Arabidopsis, *AtSHB1* and *AtIKU2* are known as positive regulatory genes for the determination of seed size/mass and are direct target genes of *AtBZR1* (Jiang et al., 2013; Li et al., 2019). In the Brachypodium genome, *BdPHO1-2(BdSHB1, Bradi3g54920)* and *BdIKU2(Bradi4g11740)* are homologs for *AtSHB1* and *AtIKU2*, respectively. RT-PCR analysis showed that the expressions of *BdPHO1-2(BdSHB1)* and *BdIKU2* were increased in the seeds of transgenic plants in comparison with wild-type seeds. Taken together, a BR signal transduction pathway *via* BdBRI1 → BdBZR1 → BdPHO1-2 (BdSHB1) → BdIKU2 seems to be operant to determine the size/mass of seeds in transgenic Brachypodium. The detailed molecular regulation of BdPHO1-2 (BdSHB1) and BdIKU2 by BR signaling *via* BdBZR1 in transgenic Brachypodium is currently under investigation.

### Seed-Specific Expression of *AtCYP85A2* Promotes Quantity and Quality of Seeds in Transgenic Brachypodium

**Figure 6:**
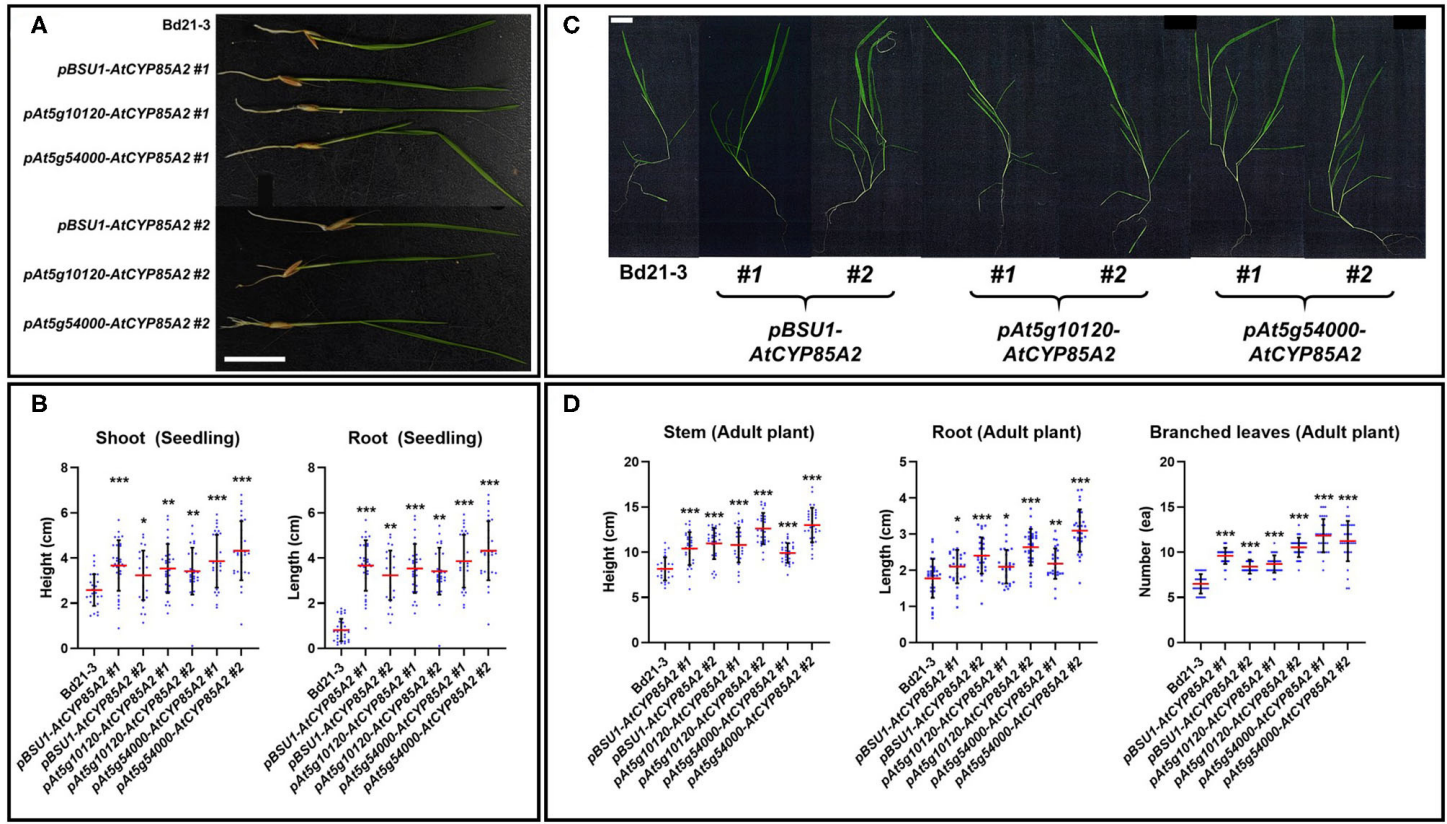
Phenotypic alterations of transgenic Brachypodium in vegetative growth. **(A)** Seedling growth of wild type and transgenic Brachypodium. Seedlings were grown in MS medium for 3 weeks. **(B)** Length of shoot and root in seedling of wild type and transgenic Brachypodium. **(C)** Phenotype of adult plants in wild type and transgenic Brachypodium. Plants were grown in soil for 6 weeks. **(D)** Length of stem, root, and number of branched leaves in wild type and transgenic Brachypodium. #1 and #2 represent two different lines for three transgenic plants. The scale bar in **(A)** and **(C)** indicates 1 cm. Each dot in **(B)** and **(D)** indicates individual data. Red and black line represents the mean of the individual measurements and the standard deviation (S.D.), respectively. The asterisks indicate the statistical significance of the Student's *t*-test: * (*P* < 0.05), ** (*P* < 0.01), and *** (*P* < 0.001).

To determine the physiological effect of the seed-specific expression of *AtCYP85A2*, phenotypic alternations in *pBSU1-AtCYP85A2::Bd21-3* (#1 and #2)*, pAt5g10120-AtCYP85A2::Bd21-3* (#1 and #2), and *pAt5g54000-AtCYP85A2::Bd21-3* (#1 and #2) were investigated. Compared to wild-type, the growth and development of shoots and roots was greater in 3-week-old seedlings of transgenic plants (Figures 6A,B). In 6-week-old plants, the stem height and number of branched leaves were also greater in transgenic Brachypodium (Figures 6C,D). These results imply that the increased endogenous levels of CS and BL in seeds may induce enhanced vegetative growth in transgenic Brachypodium.

**Figure 7:**
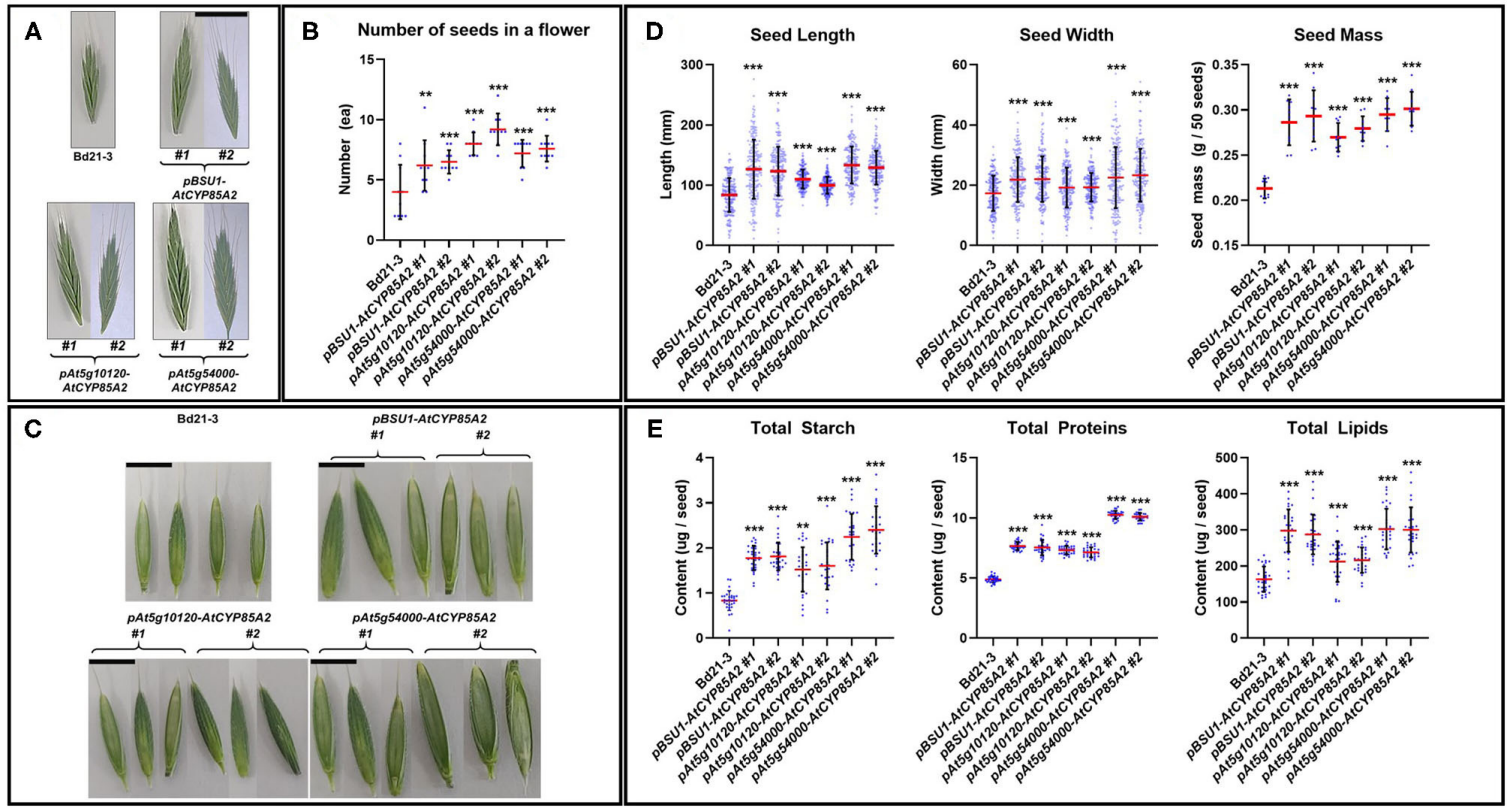
Alterations of phenotype and contents of primary metabolites in seeds of transgenic Brachypodium. **(A)** Size of flower in wild type and transgenic Brachypodium. The scale bar in **(A)** indicates 1 cm. **(B)** Number of seeds in a flower in wild type and transgenic Brachypodium. **(C)** Shape of seeds in wild type and transgenic Brachypodium. The scale bar in **(C)** indicates 50 mm. **(D)** Size and mass of seeds in wild type and transgenic Brachypodium. **(E)** Total starch, proteins, and lipids content of seeds in wild type and transgenic Brachypodium. #1 and #2 represent two different lines for three transgenic plants. Each dot in **(B,D,E)** indicates individual data. Red and black line represents the mean of the individual measurements and the standard deviation (S.D.), respectively. Asterisks indicate the statistical significance of the Student's *t*-test: ** (*P* < 0.01) and *** (*P* < 0.001).

In reproductively growing plants, the flower size and the number of seeds in flowers were remarkably greater in the transgenic lines in comparison with the wild type (Figures 7A,B). Moreover, transgenic Brachypodium showed longer and wider seeds, which resulted in the production of heavier seeds in transgenic plants compared to those in wild-type Brachypodium (Figures 7C,D). Total starch, protein, and lipid contents were also greater in the seeds of transgenic plants (Figure 7E). Thus, the seed-specific introduction of *AtCYP85A2* in *pBSU1-AtCYP85A2::Bd21-3* (#1 and #2)*, pAt5g10120-AtCYP85A2::Bd21-3* (#1 and #2), and *pAt5g54000-AtCYP85A2::Bd21-3* (#1 and #2) improved grain yield and seed quality in *B. distachyon*.

## Discussion

6-DeoxoCS and CS, but not BL, were identified from liverwort, moss, lycophyte, and fern. This implies that in non-flowering land plants, 6-deoxoCS is converted to CS by BR 6-oxidase (most likely CYP85A1), but conversion of CS to BL by BL synthase (most likely CYP85A2) does not occur in these plants (Yokota et al., 2017). In angiosperms, CYP85A1 is only active in monocotyledonous plants, whereas both CYP85A2 and CYP85A1 are active in dicotyledonous plants. This suggests that CYP85A2 activity was acquired in dicotyledonous plants after non-flowering land plants evolved to flowering land plants, angiosperms. The reason why CYP85A2 functions only in dicotyledonous plants is still unknown. In Arabidopsis, BL was not found in the shoot and leaf, but a high level of BL, which was higher than that of CS, was observed in reproductive organs such as siliques and seeds. This suggests that Arabidopsis plants require BL at particular development stages to support the maturation of reproductive organs. Therefore, the introduction of CYP85A2 as BL synthase could be related to control of reproductive development in dicotyledonous plants.

Both AtCYP85A1 and AtCYP85A2 consist of 465 amino acids. They share 83% identity and 92% similarity. However, AtCYP85A1 and AtCYP85A2 show different substrate specificities for 6-deoxoCS and CS. Chimeric enzymes constructed by exchange of *AtCYP85A1* and *AtCYP85A2* gene fragments revealed that the sterol recognition site (SRS) 1 region in the N-terminal 174 amino acids of CYP85A2 may play an important role in the recognition of C_27_- and C_28_-BRs. This finding suggests that a small number of amino acids that differ between AtCYP85A1 and AtCYP85A2, most likely in six SRSs commonly present in AtCYP85A1 and AtCYP85A2, may determine the differential substrate specificity for 6-deoxoCS and CS in BR biosynthesis (Kim et al., 2005).

In rice, BRs are involved in the control of various key agronomic traits such as plant height, leaf angle, and seed size, since they are ideal reagents for improved grain yield in monocotyledonous crops (Wu et al., 2008; Zhang et al., 2014). However, the high cost of BR synthesis discourages their direct application to crop plants (Tong and Chu, 2012). For this reason, genetic modulation of BR biosynthesis genes should be a promising approach to increase BR activity and improve grain yield in crop breeding (Shimada et al., 2003; Sahni et al., 2016; Li et al., 2018).

In BR physiology, biosynthetic precursors to CS and BL show no or much less biological activity compared to CS and BL, indicating that regulation of the endogenous level of CS and BL is important for exerting BR activity in plants. Therefore, the manipulation of genes encoding BR 6-oxidase and BL synthase to synthesize CS and BL seems particularly important in the application of BR activity in crop breeding. For this reason, in this study, AtCYP85A2 served as a bifunctional enzyme for BR 6-oxidase and BL synthase expressed in Brachypodium, which served as a model plant for monocotyledonous crop, and its efficiency in the promotion of grain yield was examined. *AtCYP85A2* driven by Arabidopsis seed-specific promoters *pBSU1, pAt5g10120*, and *pAt5g54000* was intensively expressed only in the seeds of transgenic Brachypodium, *pBSU1-AtCYP85A2::Bd21-3, pAt5g10120-AtCYP85A2::Bd21-3*, and *pAt5g54000-AtCYP85A2::Bd21-3*. The crude enzyme assay and quantitative analysis of BRs revealed that a fair amount of BL not present in the wild type was newly biosynthesized in the seeds of transgenic Brachypodium. Thus, *AtCYP85A2* possibly encodes an enzyme responsible for BL synthase (AtCYP85A2) in the seeds of transgenic Brachypodium. In most plants, BL content is 5–10 times less than CS content, even in the reproductive organs. However, the endogenous level of BL in the seeds of transgenic Brachypodium was higher than that of CS, implying that *AtCYP85A2* encodes a powerful BL synthase in the seeds of transgenic plants. Although all enzyme preparations from the seeds of transgenic plants and the wild-type catalyzed conversion of 6-deoxoCS to CS, the specific enzyme activity of BR 6-oxidase in the seeds of transgenic Brachypodium was higher than that in the wild type. Enhancement of BR 6-oxidase activity significantly reduced the endogenous level of 6-deoxoCS, while the level of CS was increased in the seeds of transgenic Brachypodium, which suggests that AtCYP85A2 also exerts effects on BR 6-oxidase in the seeds of transgenic lines. In RT-PCR, expression of a native gene for BR 6-oxidase in Brachypodium—*BdCYP85A1*—was suppressed by exogenously applied BL (Figure 5A). The reduced expression of *BdCYP85A1* was also found in a transgenic Brachypodium plants. Nevertheless, a higher endogenous level of CS was detected in the seeds of transgenic Brachypodium plants in comparison with the wild type (Table 1). These findings suggest that the reduced BR 6-oxidase activity of BdCYP85A1 is distinctly overcome by *AtCYP85A2* expressed in the seeds of transgenic Brachypodium. Thus, seed-specific introduction of *AtCYP85A2* can encode a highly active bifunctional AtCYP85A2 for both BR 6-oxidase and BL synthase in the seeds of transgenic Brachypodium.

All three seed-specific promoters in Arabidopsis, *pBSU1, pAt5g10120*, and *pAt5g54000*, successfully introduced *AtCYP85A2* to Brachypodium. This implies that the regulatory function of dicotyledonous seed-specific promoters is conserved in the monocotyledonous Brachypodium. The enzyme activities for BR 6-oxidase and BL synthase in *pAt5g54000-AtCYP85A2::Bd21-3* were higher than those in *pBSU1-AtCYP85A2::Bd21-3* and *pAt5g10120-AtCYP85A2::Bd21-3*. As a result of the increased enzyme activities, greater amounts of CS and BL were biosynthesized in the seeds of *pAt5g54000-AtCYP85A2::Bd21-3* than in the other transgenic seeds. Thus, *pAt5g54000-AtCYP85A2::Bd21-3* yielded bigger flowers with more seeds compared to those in other transgenic lines. Individual seeds in *pAt5g54000-AtCYP85A2::Bd21-3* were also more consistent in quantity and quality compared to those of *pBSU1-AtCYP85A2::Bd21-3* and *pAt5g10120-AtCYP85A2::Bd21-3*. These findings indicate that *pAt5g54000* is a more potent seed-specific promoter than *pBSU1*and *pAt5g10120* for introducing *AtCYP85A2* in Brachypodium.

In *A. thaliana*, both castasterone and brassinolide are sensed by the heterodimeric membrane-localized receptor kinases brassinosteroid insensitive 1 (AtBRI1) and AtBRI1-associated receptor kinase 1 (AtBAK1). The signal is transferred to the BR-signaling kinases (AtBSKs) and AtBRI1 suppressor 1 (AtBSU1), which suppress the kinase activity of brassinosteroid insensitive 2 (AtBIN2), a negative regulator in BR signaling. The inactivation of AtBIN2 leads to accumulation of BR transcription factors such as brassinazole resistant 1 (AtBZR1) and AtBRI1 EMS suppressor 1 (AtBES1) in the nucleus, which regulate the transcription of BR-responsive genes to exert the effects of BRs on the growth and development of plants. Homologs of BR receptors and signaling components in Arabidopsis, such as *BdBRI1, BdBAK1, BdBSKs, BdBSU1, BdBIN2*, and *BdBZR1*, are found in the Brachypodium genome, implying that similar BR signaling pathways operate in Arabidopsis and Brachypodium. As shown in Figure 5B, induction of BR signaling (*BdBRI1* and *BdBZR1*) and expression of target (*BdPHO1-2* and *BdIKU2*) genes are activated by application of BL and in transgenic Brachypodium where BL is newly synthesized. Although BL does not exist in Brachypodium, it can still act within the BR signaling pathway to regulate expression of downstream target genes in transgenic Brachypodium.

*OsDWARF4*, an upstream biosynthetic gene for rate-limiting BR biosynthesis has been intensively tested to increase BR activity in rice breeding (Li et al., 2018). Overexpression of *OsDWARF4* showed positive effects on increased grain yield, but the rate of increase was unremarkable in transgenic rice plants, even in transgenic lines in which *OsDWARF4* was seed-specifically expressed. Quantitative analysis of the biologically active BRs in transgenic rice revealed only a 1.2% increase in the endogenous level of CS, but without a concurrent increase in newly synthesized BL, by the overexpression of *OsDWARF4* compared to that in wild type, implying that a slight enhancement of BR signaling occurs by slightly increased CS to improve the grain yield of transgenic rice. Therefore, overexpression of upstream genes in BRs biosynthesis like *OsDWARF4* may not be an optimal approach to sufficiently increase BR activity in crop breeding. Although it is not native, in this study, we seed-specifically introduced the downstream gene *AtCYP85A2* for BR biosynthesis in Brachypodium. The results showed an increase of 15–27% in the endogenous level of CS in the seeds of transgenic Brachypodium (Table 1). Furthermore, considerable amounts of BL, which was absent in the wild type, were newly formed in transgenic lines. Assuming that the biological activity of BL is much higher than that of CS, these findings indicate that tens of times higher BR activity levels is generated in the seeds of transgenic lines by the expression of *AtCYP85A2*, which produces more than 200% larger seeds in transgenic Brachypodium. Therefore, for stimulating the production of biologically active CS and/or BL, introduction of the downstream gene *AtCYP85A2* in BR biosynthesis seems to be more effective than introduction of upstream biosynthetic genes that cannot synthesize BL. This technique could therefore be employed to improve seed development in monocotyledonous plants. In conclusion, the manipulation of *AtCYP85A2* in Arabidopsis for BR 6-oxidase and/or BL synthase will likely serve as an outstanding biotechnical tool for increasing BR activity and thereby increasing the grain yield in crop breeding.

## Data Availability Statement

The raw data supporting the conclusions of this article will be made available by the authors, without undue reservation.

## Author Contributions

JR and JM contributed equally to this study. JR, JM, and S-KK designed the research methodology. JR, JM, and CHP. produced transgenic Brachypodium analyzed mutants. JR, JM, and YEL performed *in vitro* crude enzyme assay and endogenous BR analysis. JR, JM, and S-KK wrote the manuscript. All authors contributed to the article and approved the submitted version.

## Conflict of Interest

The authors declare that the research was conducted in the absence of any commercial or financial relationships that could be construed as a potential conflict of interest.
