# Supplementary material for: Seed-Specific Expression of Arabidopsis AtCYP85A2 Produces Biologically Active Brassinosteroids Such as Castasterone and Brassinolide to Improve Grain Yield and Quality in Seeds of Brachypodium Distachyon
Source: Front Plant Sci. 2021 Apr 1;12:639508. doi: 10.3389/fpls.2021.639508 (PMC8047465; doi:10.3389/fpls.2021.639508)
Supplement: Supplementary file 1 [file Data_Sheet_1.pdf]

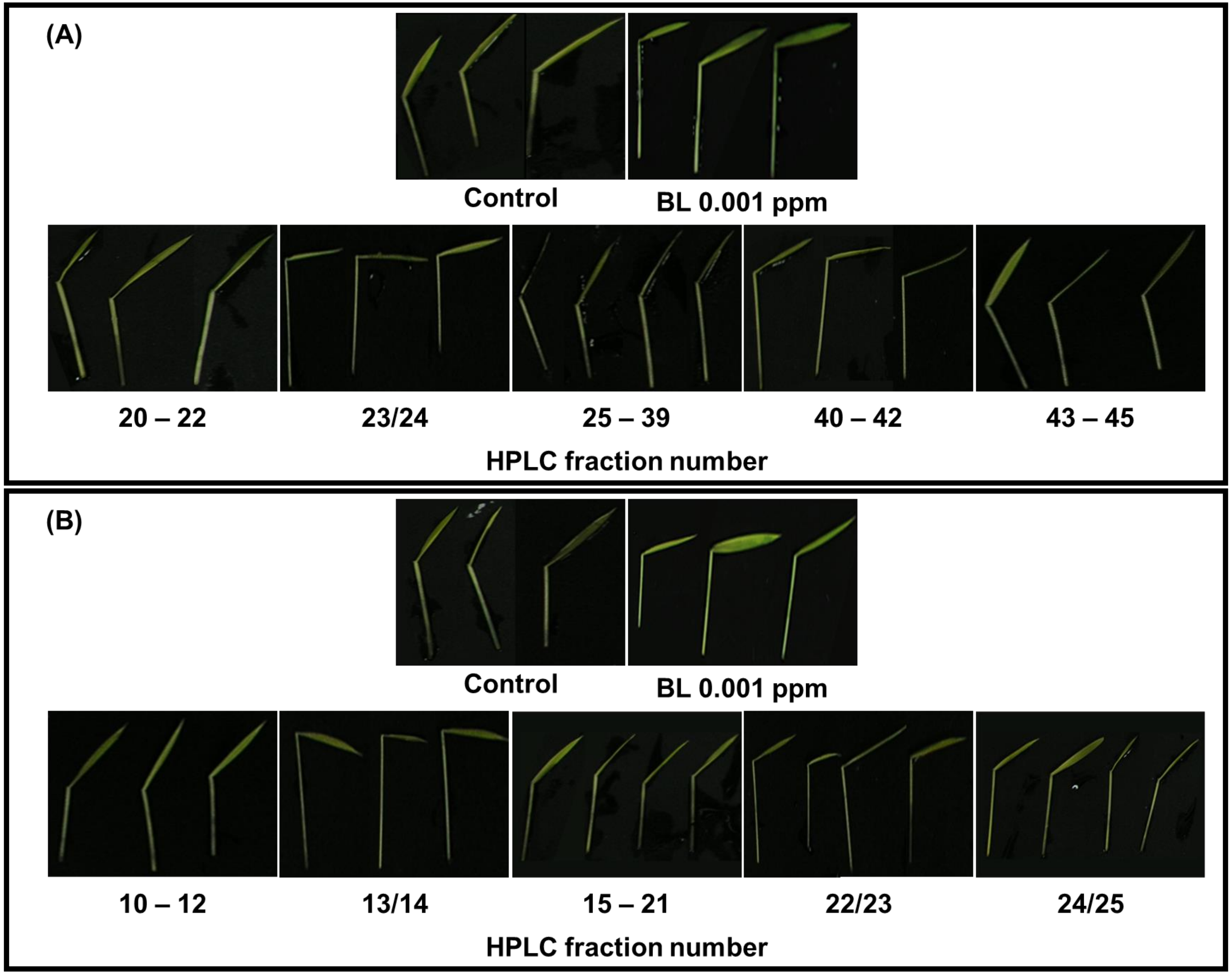

Supplementary Figure 1. BRs activity in the rice lamina inclination assay of a reversed phase HPLC
(A) Conversion of 6-deoxoCS to CS (B) Conversion of CS to BL

Supplementary table 1. The primers used for this study

Locus	Direction	Sequences	Purpose
pBSU1	For	5’-GAATTCATAATCTTGTTCATTCTTAAAAGTCCC-3’	Cloning
	Rev	5’-GGATCCAAGCAAATAATGAAGTTTAGACTC-3’	
pAt5g10120	For	5’-GGGCCCAAATTAAAGCATTAAAGTGAGACT-3’	Cloning
	Rev	5’-GGATCCTGTTTATGTTAGTCCATTATATTT-3’	
pAt5g54000	For	5’-GGGCCCATAACGGTAAAAAAAGTAGACC-3’	Cloning
	Rev	5’-GGATCCTGTTTCGGAGGATCTTTG-3’	
AtCYP85A2 (*At3g30180*)	For	5’-GGATCCATGGGCATAATGATGATGATTTTGG-3’	Cloning / semi-qPCR
	Rev	5’-GGATCCTCAGTAAGGTGAACACTTAAGATG-3’	
BdDET2 (Bradi2g55110)	For	5’-TCAACCTCCTCAACGCCTAC-3’	qRT-PCR
	Rev	5’-CGAAGTAGTTGGGACAGGTCA-3’	
BdDWF4 (Bradi1g69040)	For	5'-AGATGGAAGGGCAATGCATC-3'	qRT-PCR
	Rev	5'-AGGTGGTGCAAAAAGATGGC-3'	
BdCYP85A1 (Bradi1g15030)	For	5'-AGCGGAAATTGCAACGTTCC-3'	qRT-PCR
	Rev	5'-AACTCGGATATGTAGCCCGTTG-3'	
BdBAS1 (Bradi2g04660)	For	5'-GTGTCCGAGTGGTTCCAGAT-3'	qRT-PCR
	Rev	5'-GAGCGTCACCAGGTTCTTTC-3'	
BdSOB7 (Bradi2g44140)	For	5'-TGGACACTATGGCAAACCAA-3'	qRT-PCR
	Rev	5'-CCCATCTCGCAATCATTTCT-3'	
BdBRI1 (Bradi2g48280)	For	5'-TCTCTTTCAACCACTTCACCG-3'	qRT-PCR
	Rev	5'-GAGACTGGAGTTGGGATCTTG-3'	
BdBZR1 (Bradi1g23550)	For	5'-CTACCGCAAGGGATACAAGC-3'	qRT-PCR
	Rev	5'-GGATGGTGGTTGAGGTTGTT-3'	
BdPHO1-2 (Bradi3g54920)	For	5'-TGCCACCTCTTACCAGCTCT-3'	qRT-PCR
	Rev	5'-CAGGCCAGGCGAAGTAGTAG-3'	
BdIKU2 (Bradi4g11740)	For	5'-AAGAAGCGGAAGTCCAGTGA-3'	qRT-PCR
	Rev	5'-CTCCATCCATCTGGCCTTTA-3'	
BdGAPDH (Bradi3g14120)	For	5'-GCTCCCATGTTTGTTGTCG-3'	qRT-PCR
	Rev	5'-GACCCTCAACAATGCCAAAG-3'	
BdGAPDH (Bradi3g14120)	For	5′-ATGTTCAAGTACGACACCGTCCAC-3’	Semi-qPCR
	Rev	5′-CTACTGAGTCTTGGCCATGTGG-3′	

Supplementary table 2. Recipes of media used for callus regeneration of *Brachypodium* mutants

Media	Supplements & conditions
Callus induction media (CIM)	1X LS[a], 3% Sucrose, 0.6 mg L^{-1} $CuSO_4$, 2.5 mg L^{-1} 2,4-D[b], 0.3 % Phytagel, pH 5.8
Co-cultivation media (Co-cult)	1X MS[c], 3% Sucrose, 2.5 mg L^{-1} 2,4-D[b], 60 mg L^{-1} Acetosyringone, 0.3 % Phytagel, pH 5.8
Regeneration media (ReM)	1X MS[c], 3% Sucrose, 0.2 mg L^{-1} Kinetin, 0.3% Phytagel, 40 mg L^{-1} hygromycin, 75 mg L^{-1} Timentin[d], pH 5.8
Rooting media (RoM)	0.4X MS[c], 1% Sucrose, 0.3 % Phytagel, 0.6% Plant agar, 0.7% Charcoal, 40 mg L^{-1} hygromycin, 75 mg L^{-1} Timentin[d], pH 5.8

[a] LS : Linsmaier and Skoog medium

[b] 2,4-D : 2,4-Dichlorophenoxyacetic acid

[d] MS : Murashige and Skoog medium

[c] Timentin : Ticarcillin disodium / clavulanate potassium

Supplementary table 3. Raw data for qRT-PCR of BR metabolic genes in transgenic Brachypodium

	BdGAPDH			*BdDET2*			*BdDWF4*			*BdCYP85A1*			*BdBAS1*			*BdSOB7*		
	Ex-1	Ex-2	Ex-3	Ex-1	Ex-2	Ex-3	Ex-1	Ex-2	Ex-3	Ex-1	Ex-2	Ex-3	Ex-1	Ex-2	Ex-3	Ex-1	Ex-2	Ex-3
Bd21-3	23.87	23.41	23.44	26.51	26.77	25.94	27.12	26.97	27.28	26.33	25.01	25.87	25.12	24.96	25.02	26.02	25.98	25.7
BL treatment	23.29	23.72	23.95	26.99	27.44	26.25	27.69	27.62	28.16	26.92	25.87	26.59	24.79	24.43	24.39	25.78	25.55	25.19
pBSU1-AtCYP85A2 #1	23.17	23.38	23.52	27.03	27.29	26.24	27.16	27.16	27.63	26.65	25.24	26.05	23.81	23.89	23.83	25.48	25.24	24.76
pBSU1-AtCYP85A2 #2	23.51	23.87	23.41	27.32	27.43	26.67	27.44	27.5	27.91	27.12	25.53	26.24	24.36	24.05	24.01	25.39	25.73	25.24
pAt5g10120-AtCYP85A2 #1	23.53	23.29	23.51	26.77	27.19	26.28	27.12	27.21	27.86	26.76	25.33	26.08	24.32	23.88	23.99	25.59	25.72	24.95
pAt5g10120-AtCYP85A2 #2	23.44	23.17	23.38	26.64	26.96	26.22	27.06	27.14	27.74	26.46	25.71	25.93	24.19	23.72	23.93	25.38	25.51	24.89
pAt5g54000-AtCYP85A2 #1	23.95	23.51	23.72	27.3	27.72	27.65	27.68	27.84	28.25	27.59	26.14	26.82	24.38	24.09	24.03	25.75	25.55	25.1
pAt5g54000-AtCYP85A2 #2	23.52	23.44	23.46	27.14	27.99	26.91	27.39	27.79	27.91	27.36	25.58	26.26	24.22	23.74	23.91	25.32	25.51	24.87

Supplementary table 4. Raw data for qRT-PCR of BR signaling and seed size-determining genes in transgenic Brachypodium

	BdGAPDH			*BdBRI1*			*BdBZR1*			*BdPHO1-2 (BdSHB1)*			*BdIKU2*		
	Ex-1	Ex-2	Ex-3	Ex-1	Ex-2	Ex-3	Ex-1	Ex-2	Ex-3	Ex-1	Ex-2	Ex-3	Ex-1	Ex-2	Ex-3
Bd21-3	23.87	23.41	23.44	25.21	24.38	24.03	26.13	25.45	25.99	19.31	18.33	18.89	18.89	18.23	18.36
BL treatment	23.29	23.72	23.95	24.19	23.32	22.77	25.63	24.85	25.27	18.99	17.74	18.42	18.58	17.81	18.17
pBSU1-AtCYP85A2 #1	23.17	23.38	23.52	23.66	22.9	22.32	25.06	24.51	24.79	18.59	17.77	18.03	18.07	17.8	17.77
pBSU1-AtCYP85A2 #2	23.51	23.87	23.41	23.95	22.91	22.66	25.28	24.56	25.06	18.87	17.77	18.57	18.62	17.75	17.81
pAt5g10120-AtCYP85A2 #1	23.53	23.29	23.51	23.92	23.25	22.62	25.3	24.5	24.91	18.99	17.79	18.21	18.38	17.94	17.67
pAt5g10120-AtCYP85A2 #2	23.44	23.17	23.38	23.96	23.01	22.52	25.07	24.35	24.84	18.96	17.5	18.35	18.27	17.82	17.58
pAt5g54000-AtCYP85A2 #1	23.95	23.51	23.72	23.91	23.1	22.7	25.5	24.68	25.22	18.9	18.01	18.29	18.57	18.11	17.87
pAt5g54000-AtCYP85A2 #2	23.52	23.44	23.46	23.71	22.7	22.43	24.96	24.35	24.96	18.37	17.58	18.23	18.14	17.64	17.89

Supplementary table 5. Raw data of GC-MS for endogenous BR analysis

Sample	Compound	*m/z*	Retention time / Area						Sample	Compound	*m/z*	Retention time / Area					
			Ex-1		Ex-2		Ex-3					Ex-1		Ex-2		Ex-3	
Bd21-3	D6-6-deoxoCS	504	10.961	4142	10.903	3202	10.966	5133	*pBSU1-AtCYP85A2*	D6-6-deoxoCS	504	10.974	4682	10.955	7061	11.048	5331
		489		1208		933		1498			489		1633		2441		1847
	D0-6-deoxoCS	498	11.07	7913	11.008	5689	11.074	9119		D0-6-deoxoCS	498	11.085	3783	11.061	6278	11.160	4387
		483		2308		1658		2659			483		1311		2173		1519
		343		1155		830		1331			343		799		1326		928
		273		16502		11858		19013			273		7277		12076		8437
		155		13697		9840		15781			155		6619		10989		7673
	D3-CS	515	14.997	117618	14.912	101806	15.003	171384		D3-CS	515	15.013	21868	14.988	22317	15.121	20309
		158		1470221		1470221		1470221			158		273369		278966		253861
	D0-CS	512	15.061	59749	14.977	56297	15.067	91347		D0-CS	512	15.072	13383	15.049	14818	15.182	12472
		357		119503		112593		182699			357		28440		31488		26505
		327		104567		98517		159855			327		23420		25933		21819
		287		209123		197033		319713			287		58551		64827		54569
		155		497913		469142		761228			155		167288		185225		155913
	D3-BL	531	19.580	632074	19.387	638614	19.504	629897		D3-BL	531	19.594	3142	19.484	3831	19.657	2613
		158		12641492		12787213		12700102			158		52367		63852		43548
	D0-BL	528	-	-	-	-	-	-		D0-BL	528	19.655	2586	19.565	3507	19.738	2355
		374		-		-		-			374		14221		19289		12949
		332		-		-		-			332		21121		28643		19233
		177		-		-		-			177		30599		41503		27864
		155		-		-		-			155		43094		58452		39253
pAt5g10120-AtCYP85A2	D6-6-deoxoCS	504	10.949	6981	10.91	5317	10.994	6276	*pAt5g54000-AtCYP85A2*	D6-6-deoxoCS	504	10.955	7185	10.974	3386	10.798	4095
		489		2583		14352		2326			489		24431		1149		1401
	D0-6-deoxoCS	498	11.056	9124	11.017	6437	11.100	8361		D0-6-deoxoCS	498	11.063	3822	11.083	1720	10.907	1990
		483		3381		2384		3097			483		1294		581		676
		343		2705		1906		2476			343		762		342		397
		273		16891		11917		15482			273		7643		3445		3981
		155		15879		11202		14554			155		6808		3061		3543
	D3-CS	515	14.983	54613	14.924	64258	15.037	79194		D3-CS	515	14.991	83388	15.016	58446	14.777	44537
		158		606811		713998		897839			158		823197		572951		439129
	D0-CS	512	15.048	32057	14.989	41253	15.103	48786		D0-CS	512	15.052	57552	15.079	37230	14.841	31528
		357		74813		96251		113835			357		115103		74463		63057
		327		49862		64173		75889			327		92089		59569		50443
		287		131797		169592		200566			287		207187		134026		113499
		155		356188		458366		542063			155		575521		372302		315277
	D3-BL	531	19.568	2474	19.401	2168	19.548	2676		D3-BL	531	19.573	2871	19.521	2948	19.21	4286
		158		82453		72281		89313			158		57613		61321		86477
	D0-BL	528	19.631	1811	19.486	1675	19.634	2162		D0-BL	528	19.638	4073	19.603	4538	19.293	5869
		374		17511		16197		20897			374		26069		29042		37564
		332		25354		23451		30265			332		38284		42658		55172
		177		41659		38523		49720			177		58655		65379		84511
		155		60364		55839		72629			155		81468		90758		117377

Supplementary table 6. Raw data of GC-MS for crude enzyme assay

Sample	Compound	*m/z*	Retention time / Area						Sample	Compound	*m/z*	Retention time / Area					
			Ex-1		Ex-2		Ex-3					Ex-1		Ex-2		Ex-3	
Bd21-3 Shoot/Root	D3-CS	515	14.071	264910	13.937	246629	14.134	287406	Bd21-3 Seed	D3-CS	515	14.099	326894	14.157	336287	14.026	298561
		158		3784428		3523271		4105811			158		3060805		3252292		2975493
	D0-CS	512	14.133	725061	13.998	719913	14.197	791231		D0-CS	512	14.162	808512	14.218	914109	14.087	783124
		357		1370242		1360511		1495303			357		1623231		1835328		1571262
		327		1081447		1076769		1180139			327		1722898		1947919		1668799
		287		2384099		2367219		2601677			287		3087984		3491292		2991014
		155		6144585		6100961		6705349			155		8169265		9236223		791344
	D3-BL	531	18.292	51394	18.118	49823	18.374	66794		D3-BL	531	18.321	78331	18.404	69138	18.234	74986
		158		1284887		1245583		1669991			158		2061343		1868594		1922718
	D0-BL	528	-	-	-	-	-	-		D0-BL	528	-	-	-	-	-	-
		374		-		-		-			374		-		-		-
		332		-		-		-			332		-		-		-
		177		-		-		-			177		-		-		-
		155		-		-		-			155		-		-		-
pBSU1-AtCYP85A2 Shoot/Root	D3-CS	515	13.999	411354	14.058	393317	13.924	409253	*pBSU1-AtCYP85A2* Seed	D3-CS	515	14.023	315473	14.104	275315	14.123	334619
		158		3344341		3075671		3459421			158		3568691		4048633		4920868
	D0-CS	512	14.058	1289199	14.119	1150837	13.981	1163517		D0-CS	512	14.089	934121	14.172	913889	14.191	1043669
		357		2445902		2183398		2207443			357		2275224		2225943		2542047
		327		2179376		1954577		1966931			327		1714170		1677051		1915230
		287		4036287		3603098		3642801			287		3704507		3624272		4138955
		155		1096873		9791023		9897943			155		9690052		9480177		10826442
	D3-BL	531	18.202	64978	18.275	62184	18.101	60843		D3-BL	531	18.232	16531	18.335	19338	18.36	17004
		158		1547123		1494808		1401912			158		311905		379176		349158
	D0-BL	528	-	-	-	-	-	-		D0-BL	528	18.316	357882	18.424	318759	18.448	315477
		374		-		-		-			374		2937462		2616431		2589408
		332		-		-		-			332		3885944		3461143		3425506
		177		-		-		-			177		7287059		6490455		6423629
		155		-		-		-			155		9894443		8812798		8722075
pAt1g10120-AtCYP85A2 Shoot/Root	D3-CS	515	14.046	354132	14.018	379510	13.937	302376	*pAt1g10120-AtCYP85A2* Seed	D3-CS	515	14.083	651374	13.961	700139	13.933	673984
		158		4599116		4928701		3929961			158		4861375		5225111		5029731
	D0-CS	512	14.105	978896	14.085	1146522	14.003	867557		D0-CS	512	14.144	2026441	14.020	2006538	13.995	2071331
		357		1866371		2185971		1654092			357		3532794		3498108		3611053
		327		1610792		1885955		1427075			327		2966656		2937521		3032284
		287		3102798		3634122		27494897			287		5888173		5830324		6018603
		155		8624636		10111634		7643675			155		18274335		18094855		18679151
	D3-BL	531	18.260	132012	18.223	129433	18.118	143273		D3-BL	531	18.311	43866	18.149	39731	18.113	44310
		158		6557973		6429588		7117945			158		719111		673406		714909
	D0-BL	528	-	-	-	-	-	-		D0-BL	528	18.390	421596	18.226	388699	18.1935	455752
		374		-		-		-			374		2867048		2643354		3099321
		332		-		-		-			332		4238458		3907733		4581842
		177		-		-		-			177		7697208		7096599		8320507
		155		-		-		-			155		9781810		9018541		10574292

pAt5g54000-AtCYP85A2 Shoot/Root	D3-CS	515	14.102	500314	14.166	475638	14.241	465174	*pAt5g54000-AtCYP85A2* Seed	D3-CS	515	14.078	273147	14.033	316438	13.990	386612
		158		3896526		3854440		3751403			158		1982198		2556042		3100336
	D0-CS	512	14.163	1567133	14.228	1361286	14.304	1399691		D0-CS	512	14.137	965820	14.096	994905	14.055	1299844
		357		3577407		3107511		3195177			357		1729328		1781405		2327407
		327		3088778		2683066		2758755			327		1251696		1289394		1684591
		287		5263395		4572035		4701022			287		2660843		2740976		3581082
		155		14673530		12746124		13105726			155		8796175		9061062		11838288
	D3-BL	531	18.336	90846	18.416	100234	18.513	89751		D3-BL	531	18.304	76811	18.243	89110	18.187	104381
		158		2064681		2078045		2039725			158		1824489		2123544		2327319
	D0-BL	528	-	-	-	-	-	-		D0-BL	528	18.381	198968	18.325	227822	18.272	283858
		374		-		-		-					3959533		3846727		4792881
		332		-		-		-					4470912		5119274		6378432
		177		-		-		-					7305885		8365377		10422966
		155		-		-		-					9169032		10478710		13080144

Supplementary table 7. Raw data of substance quantification in transgenic Brachypodium seeds

**Starch**	UV length 570 nm					
**Standard**	y = 34.036x + 0.011, R^2 = 0.9887					
Concentration (μg/μL)	0	0.008	0.016	0.024	0.032	0.04
Absorbance	0.02	0.32	0.53	0.81	1.02	1.45
**Bd21-3**						
Absorbance	0.20	0.16	0.10			
pBSU1-AtCYP85A2		**#1**			**#2**	
Absorbance	0.34	0.29	0.31	0.29	0.33	0.34
pAt5g10120-AtCYP85A2		**#1**			**#2**	
Absorbance	0.31	0.22	0.28	0.24	0.28	0.33
pAt5g54000-AtCYP85A2		**#1**			**#2**	
Absorbance	0.43	0.34	0.41	0.38	0.40	0.48
**Proteins**	UV length 595 nm					
**Standard**	y = 0.1681x + 0.3177, R^2 = 0.988					
Concentration (μg/μL)	0	0.25	0.5	1	2	
Absorbance	0.306	0.364	0.423	0.470	0.656	
**Bd21-3**						
Absorbance	0.487	0.481	0.478			
pBSU1-AtCYP85A2		**#1**			**#2**	
Absorbance	0.569	0.576	0.581	0.584	0.568	0.561
pAt5g10120-AtCYP85A2		**#1**			**#2**	
Absorbance	0.571	0.563	0.558	0.555	0.566	0.551
pAt5g54000-AtCYP85A2		**#1**			**#2**	
Absorbance	0.668	0.662	0.656	0.659	0.651	0.661
**Lipids**	UV length 530 nm					
**Standard**	y = 0.0147x + 0.1771, R^2 = 0.993					
Concentration (μg/μL)	0	1.25	2.5	5	10	
Absorbance	0.178	0.19	0.214	0.258	0.321	
**Bd21-3**						
Absorbance	0.224	0.22	0.231			
pBSU1-AtCYP85A2		**#1**			**#2**	
Absorbance	0.257	0.262	0.275	0.258	0.255	0.272
pAt5g10120-AtCYP85A2		**#1**			**#2**	
Absorbance	0.241	0.248	0.23	0.242	0.246	0.234
pAt5g54000-AtCYP85A2		**#1**			**#2**	
Absorbance	0.256	0.273	0.269	0.255	0.266	0.275
